# Decision time is associated with future cooperation and social rewiring decisions in network public goods games

**DOI:** 10.1371/journal.pone.0347919

**Published:** 2026-04-28

**Authors:** Zeyu Zhu

**Affiliations:** 1 Human Nature Lab, Yale University, New Haven, Connecticut, United States of America; 2 Department of Sociology, Yale University, New Haven, Connecticut, United States of America; Virginia Commonwealth University, UNITED STATES OF AMERICA

## Abstract

Previous research suggests that encouraging cooperation in social networks by engaging cooperators with defectors is only effective when individual heterogeneity is considered. However, this heterogeneity remains largely underexplored. Drawing on the drift-diffusion model, decision time captures this heterogeneity as an indicator of the feeling of conflict when individuals make cooperation decisions. Using secondary data from a previous public goods game experiment, this study first investigates how one’s decision time predicts one’s cooperation decision in the next round. A typical participant with a longer decision time is more likely to flip their decision. I then explore the relationship between this decision time and one’s willingness to connect to others when offered a chance. Faster defectors are more willing to connect to both cooperators and defectors than slower defectors. Faster cooperators are less willing to connect to defectors than slower cooperators, but no significant difference is found between faster and slower cooperators when offered to connect to fellow cooperators after controlling for environmental factors. The findings may serve as a foundation for transparent intervention strategies that facilitate cooperation by selectively engaging cooperator–defector pairs.

## Introduction

Cooperation is fundamental to human sociality, but for a long time it remained a puzzle why humans cooperate when defection should be rationally preferred [1]. Evolutionary game theory suggests that by choosing to cooperate, the payoff can be maximized in the long term if there are repeated interactions [2], but even when this is the case, defections can still arise.

Using the network public goods game as a model, it has been revealed that a healthy amount of social fluidity for people to rewire their social networks is conducive to the maintenance of cooperation in a society because it gives cooperators chances to disengage from defectors [3,4]. Following this line of research, a “social planner” based on reinforcement learning has been shown to successfully facilitate cooperation by selectively encouraging links to be made between cooperators and defectors [5]. This highlights the behavioral heterogeneity in people’s responses to social rewiring chances. However, due to the “black box” nature of the reinforcement learning model, it remains poorly understood what can predict one’s reactions to offers to connect to another.

Drawing from the literature on the drift-diffusion model (DDM) [6,7], this study attempts to probe into this heterogeneity by using decision time as a real-time measure of individuals’ mental conflict [8–10]. It finds that *current* cooperation decision time is predictive of *future* cooperation and social rewiring decisions.

### Social rewiring in public goods games

One popular model to understand human cooperation behavior is the public goods game [11,12]. A simplified version is as follows: in a group of people, each player has to decide whether to cooperate or to defect. To cooperate means that the player has to contribute a certain amount of money, and this amount will be doubled and equally divided among other players. To defect means that the player contributes nothing and only expects to receive the benefit of the doubled contributions from other players. The rational behavior that maximizes payoff is to defect, i.e., to freeride. Typically, the game is run for multiple rounds to illicit reciprocity.

Based upon this model, a network public goods game is developed to understand human cooperation in social network contexts [3,13]. It replicates the classical public goods game, but instead of being situated in an amorphous group, each player in the network version of this game only interacts with their neighbors in the social network. Therefore, players do not simultaneously interact with all the other players in a session, but only interact with a subset of them.

The network public goods game captures two mechanisms that govern real-world human behaviors: social loafing and social contagion [14]. First, in larger groups, people feel that their contribution is less likely to make a difference to the common good and therefore have a greater tendency to free ride [14,15]. This mechanism has been confirmed in experimental settings where people with more neighbors are more likely to defect [4]. Second, cooperation is known to cascade in social networks just as many other things do [16]. When there are multiple sources of contagion, such a contagion can be more likely to happen [17]. In other words, one’s social environment matters. Empirically, both the number of cooperative neighbors and the proportion of cooperative neighbors can increase one’s likelihood to cooperate [4,14].

Notably, this network need not be static, and some studies have allowed the rewiring of ties in the network [e.g., 3–5]. To be specific, a fraction of all dyads are randomly chosen after each round. For each dyad, if a tie exists within it, then one of the two nodes will be randomly picked and given the option to break this tie (subjects are typically informed of the cooperation behavior of candidate alters); otherwise, if both nodes agree, the tie will be established. It is found that this fluidity in the network is conducive to the maintenance of global cooperation rate in the session, without which defection would overwhelm the entire network over time [3].

Following this line of research, Shirado and Christakis [4] show that giving people extra opportunities to break their ties with their neighbors (“disengagement strategy”) can increase the global cooperation level in a network group. Meanwhile, randomly encouraging connections between defectors and cooperators (“engagement strategy”), in hope of changing the defectors’ behavior, turns out to be detrimental to the level of cooperation because the harm a new defector neighbor causes to a cooperator typically outweighs the benefit it brings to the defector [4].

To overcome this issue, McKee et al. [5] uses a deep reinforcement learning model to strategically plan the engaging and disengaging processes by taking into account the history of the players’ behavior, and this model is shown to be effective in encouraging cooperation. A post-hoc examination of this “reinforcement learning planner” shows that it tends to engage defectors and cooperators in earlier rounds and tends to disengage them in later rounds.

This reveals critical individual heterogeneity in people’s responses to social rewiring and their upcoming cooperation decisions, as evidenced by the planner’s strategic rewiring behavior. Nevertheless, this heterogeneity remains underexplored. In McKee et al. [5], due to the black box nature of the deep learning model, the interpretability of the strategy employed by the model is lacking. To better understand this heterogeneity, the present study takes a step back from an interventionist’s perspective and focuses on decision time as a real-time measure of the individual’s internal mental state.

### Decision time as a measure of mental conflict

Decision time can provide valuable information about one’s mental conflict when making the decision—a longer decision time implies higher mental conflict [8]. As a result, people can use others’ decision time to understand their underlying mental state [18–20]. In some scenarios, people may even strategically use or manipulate their own decision time as a signal to others [21,22].

The drift-diffusion model (DDM) [6,7] offers a theoretical account of the human decision making process, in which decision time is a byproduct. Suppose there are two options in the decision. DDM views decision making as a sequential sampling process—at every moment, the decision maker accumulates evidence supporting either option, contributing to a signal that determines which option to favor. Over time, the accumulated signal drifts between two thresholds; crossing a threshold will lead the decision maker to choose the corresponding option. Naturally, with more conflicting evidence, there will be more fluctuation in the signal, and it will take longer before a threshold can be reached. Therefore, decision time can be used as a measure of internal conflict experienced by the decision maker.

Formally, the decision maker’s internal evidence state X(τ) evolves continuously over decision time τ as:

$\text{d}X=v_{t}\text{d}\tau +\sigma \text{d}W,$where $v_{t}$ is the drift rate in round t, σ is a noise parameter, and dW is Gaussian white noise [23]. The process starts at a neutral point and terminates when X reaches one of the two thresholds. The drift rate $v_{t}$ is the difference in the expected utilities of the two options:

$v_{t}=u_{t}(C)-u_{t}(D),$where $u_{t}(C)$ and $u_{t}(D)$ are the decision maker’s (subjective) expected utilities of the two options (e.g., cooperation and defection in the public goods game) in round t. When $v_{t}>\text{0}$, evidence drifts towards option C; when $v_{t}<\text{0}$, evidence drifts towards option D.

Given these definitions, the expected decision time ${\text{RT}}_{t}$, i.e., the time τ at which X reaches a threshold, is a monotonically decreasing function of $\left|v_{t}\right|$. Therefore, the observed decision time can be used as a proxy for one’s preference. A long decision time implies that the decision maker is close to indifference, i.e., great internal conflict when making the decision, and a short decision time implies that the decision maker has a clear preference, i.e., low internal conflict.

Empirical evidence has supported DDM by linking decision time and various factors related to mental conflict in different scenarios. In scenarios where there is an objectively correct answer, e.g., whether more points are moving left or right on a screen, it is shown that people react faster when the task is easier [24]. In other scenarios where the decision is based on the respondent’s preference, the speed of decision has been linked with visual fixation on choices—as the respondent looks at one of the options, the signal drifts towards that option, and the resulting decision time can be well predicted by DDM [6,23,25].

In public goods games, it is found that higher extremity of decisions, supposedly related to lower mental conflict, is predicted by shorter decision time [8,9]. Furthermore, people are faster deciders when their social environment reciprocates their own decisions [10]. While some of these studies are not explicitly based on DDM, their findings are consistent with DDM’s predictions.

Despite the efforts devoted to understanding the role of decision time in public goods games, decision time has been overwhelmingly studied as an outcome variable [8–10,26–28]. The present study takes a step further by exploring the potential of *current* decision time to be used as a predictor of the participants’ *future* behaviors, including cooperation decisions and rewiring choices, given its signifying role of the feeling of conflict.

To explicate, recall the settings of the network public goods games [3,4,29,30]: In these games, a pair of participants (dyad) has the option to make or break the ties between them if the dyad is randomly chosen. For a dyad that is currently connected by a tie, one of the two participants will be randomly picked and offered the option to break the tie. If this participant agrees, the tie will be removed from the network. If the dyad is not currently connected, the establishment of a new tie will require both participants’ agreement, and both participants can see the (cooperation or defection) decision that is just made by the other participant.

In this process, it is theoretically predicted and empirically observed that defectors will likely seize the chance to connect to cooperators when offered, but cooperators will be reluctant to connect to defectors [3,30]. Despite this overall tendency, there may exist heterogeneity in each group that can be predicted by the time used to make the cooperation decision. Here, I systematically consider all four scenarios when a pair is offered a chance to connect: (a) defectors offered a chance to connect to a defector, (b) defectors offered a chance to connect to a cooperator, (c) cooperators offered a chance to connect to a defector, and (d) cooperators offered a chance to connect to a cooperator.

For defectors, if faster ones experience less conflict in their free riding, they may be more willing to maximize their benefit by connecting to others, whether they are being connected to a cooperator or a defector. Connecting to a cooperator makes intuitive sense—if the cooperator keeps cooperating, the defector’s payoff will increase. Connecting to a fellow defector is also desirable because adding such a connection has no cost but their defector neighbors may turn to cooperation in the future. Meanwhile, for slower defectors who are experiencing greater mental conflict, connecting to fellow defectors will make it harder for them to turn to cooperation in the future, which they are more likely to be considering, and connecting to cooperators, although helpful for turning their social environments more cooperative, will likely require them to hurt the cooperators because they may need to keep defecting to protect themselves from the exploitation of their social environments, making them hesitant to accept the offer as well.

For cooperators, a similar heterogeneity may also be expected, although it is essentially an empirical question as to the direction of the relations. It is difficult to predict how cooperators will respond when offered a chance to connect to others, no matter they are cooperators or defectors. On the one hand, faster cooperators who experience less mental conflict may be more protective of their current social environment and display greater risk aversion, which may cause them to shield off any new neighbors, including current cooperating ones. On the other hand, they may also be more benign people in general and are more willing to connect to people, even when they are not cooperating.

After the rewiring process in such games, the session proceeds to the next round and the participants make cooperation decisions again with their (potentially) updated neighbors. Decision time can also be useful to predict the cooperation decision next round when considered together with the current-round decision. To be specific, one can expect those who are experiencing greater mental conflict and exhibit longer decision time to be more likely to flip their decisions in the next round.

Formally, an assumption about incremental environmental change needs to be made. Between rounds, the drift rate is assumed to change by a stochastic perturbation:


$$v_{t+\text{1}}=v_{t}+\epsilon_{t+\text{1}},$$

where $\epsilon_{t+\text{1}}$ is a random variable that reflects the changes in the decision maker’s environment, the round index (considering the possible learning/fatigue effects), and other random factors.

Without loss of generality, consider the probability of a cooperator in round t flipping to defection in the next round:


$$\text{Pr}\left(flip|v_{t}>\text{0}\right)=\text{Pr}\left(\epsilon_{t+\text{1}}<-v_{t}\right)=F\left(-v_{t}\right),$$

where F is the cumulative density function (CDF) of $\epsilon_{t+\text{1}}$. Since the CDF is a monotonically increasing function, this probability decreases with $v_{t}$. This means that a cooperator with a lower $v_{t}$, as reflected by a longer decision time, has a higher probability of flipping next round.

To summarize, the research questions this study hopes to answer are as follows:

H1: Longer current-round decision time predicts a higher probability of flipping the decision in the next round.

H2: For defectors, decision time is negatively related to one’s willingness to accept an offer to connect to a defector.

H3: For defectors, decision time is negatively related to one’s willingness to accept an offer to connect to a cooperator.

RQ1: For cooperators, is there a relation between decision time and one’s willingness to accept an offer to connect to a defector? If so, what is the direction of this relation?

RQ2: For cooperators, is there a relation between decision time and one’s willingness to accept an offer to connect to a cooperator? If so, what is the direction of this relation?

### Data

The present study re-analyzed publicly available data from the Nishi et al. [29] experiment, supplemented by previously unavailable data useful for present purposes; the original intent of that experiment was to understand how economic inequality within a network and the visibility of this inequality could change cooperation outcomes. It turned out that the visibility of neighbors’ wealth alone was detrimental to the global cooperation rate regardless of the initial distribution of wealth (even when there was no initial inequality), and the initial distribution of wealth was relatively unimportant. Their experiment created four initial distributions of wealth and, for each distribution, included 10 sessions for the visible condition and the invisible condition, respectively, resulting in a total of 80 sessions. On average, 17.21 participants (s.d. = 7.13) participated in each session. At the start of each session, 30% of the dyads were randomly sampled and connected by a tie. Each session lasted for 10 rounds. The dataset included 1,462 participants, who made a total of 13,560 decisions.

In each round, participants could choose to cooperate by giving 50 units to each neighbor, who would receive 100 units, or to defect by giving 0 units. After this economic transaction, 30% of possible dyads were sampled. Then, the game proceeded in the same way as introduced above.

A participant’s decision time in a given round was obtained by comparing two timestamps: one recorded when the round started, the other recorded when a cooperation decision was made. Both timestamps were automatically recorded by Breadboard [31], the software platform that enabled the original online experiment. Although the original study [29] did not exploit this variable, two studies have explored the role of decision time with this dataset [10,28]. One study [28] found that it was faster for U.S. participants to cooperate than to defect, but it was faster for Indian participants to defect than to cooperate. The other study [10], synthesizing this dataset and others, found that people were faster deciders when their social environments reciprocated their decisions. These studies, however, viewed decision time as an outcome of the participant’s behavior in the same round, while I used decision time as a predictor of the participant’s future behavior.

Following existing research [e.g., 10,32], the natural log of the decision time (in seconds) was used in the statistical models. The log-decision time had a mean of 1.47, a median of 1.34, and a standard deviation of 0.68 (N = 13,560). Over the rounds, the participants spent less time making the cooperation decision, which could be because they were getting more familiar with the game, or their social environment became more stabilized so that it was easier for them to decide to reciprocate [10], or both. See S1 Appendix A for visual inspections of the distributions regarding decision time.

### Ethics statement

This study involved the secondary analysis of an existing dataset [29]. The research protocol was reviewed and granted an Exemption Determination by the Yale University Institutional Review Board (IRB Protocol ID: 2000034830; Modification ID: MOD00067863). As the study was determined to be exempt research involving secondary analysis of existing data, the requirement for informed consent was waived.

## Results

### Slow deciders flip

**Fig 1:**
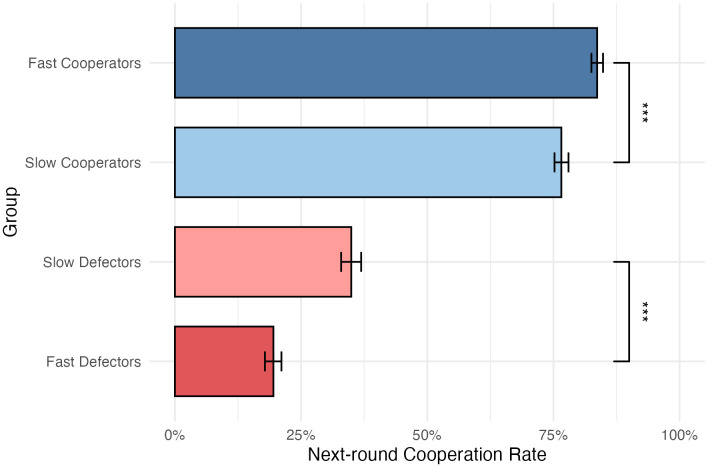
Next-round cooperation rates among fast cooperators, slow cooperators, slow defectors, and fast defectors. Error bars show 95% CIs of means. ***: *p* < 0.001.

The first goal of this study is to confirm the prediction that slower deciders are more likely to flip their decisions in the next round (H1). To start with, I categorized each participant–round (N = 13,560) by decision (cooperation or defection) and decision time (within each decision category, top 50% were considered “slow” and bottom 50% were considered “fast”). Fig 1 compares the next-round cooperation rates of the four groups. Slow cooperators were more likely to defect in the next round than fast cooperators (*p* < 0.001, two-tailed t-test), and slow defectors were more likely to cooperate in the next round than fast defectors (*p* < 0.001, two-tailed t-test). In general, slower deciders were more likely to change their decisions. This is consistent with the theory of decision time as an indicator of feeling of conflict.

Meanwhile, other factors might have confounded these relationships. For instance, round index can predict both speed and cooperation. Therefore, I used a generalized linear mixed effects model (GLMM) with a logistic link function (Model 1) to predict the next-round decision, controlling for current-round decision, decision time, and the number of accepted offers to connect to cooperators and defectors (S1 Appendix B), the two-way interactions between decision and the above variables, the round index. I also controlled for other known predictors [4,5,29], including the participants’ current-round environmental factors (namely the number of neighbors, the number of cooperative neighbors, and the proportion of cooperative neighbors) and the visibility of neighbors’ wealth. In this and all subsequent models, cooperation decisions were binary, with cooperation encoded as 1 and defection as 0. Decision time was log-transformed and centered. Preliminary analysis showed that a random intercept for session was unnecessary, consistent with Shirado and Christakis [4]’s result. Therefore, only a random intercept was used for participant to account for overall individual heterogeneity in baseline willingness to cooperate. This helped rule out the potential issue of regression to the mean. This model accommodated both cooperators and defectors. The specification of the model was also partly informed by an analysis of the participants’ next-round decisions facing environmental changes. See S1 Appendix B for details.

**Fig 2:**
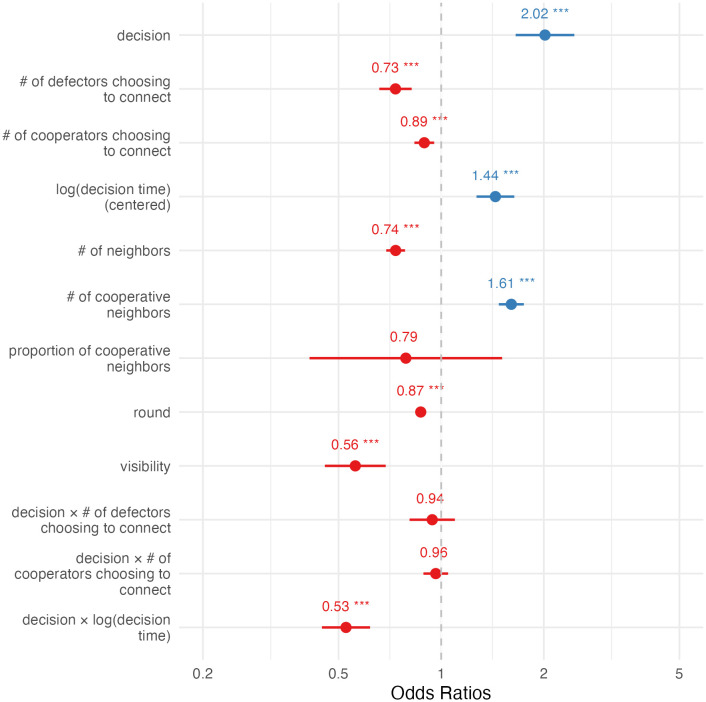
Estimated fixed effects of Model 1, predicting next-round cooperation (cooperate = 1). Error bars show 95% CI.

The result (Fig 2; S1 Appendix C) agreed with the simplified picture shown above (even after controlling for round and including a random intercept for participant). For a typical defector, a one-unit increase in the log-decision time was linked with a 44% (95% CI = 27%, 64%) increase in the odds of cooperating next round. For a typical cooperator, this was related to a decrease in the odds of next-round cooperation by 24% (95% CI = 15%, 32%; the CI was derived using (co)variances obtained from the covariance matrix estimated by the model: var(A)=0.0042;var(B)=0.0069;cov(A, B)=−0.0039, where A and B are log-decision time and the interaction between decision and log-decision time). I also considered an expanded model where the effects could change over the rounds. The results (S1 Appendix C) corroborated the above findings. To summarize, this confirmed the hypothesis that slower deciders were more likely to flip.

### Fast defectors connect, fast cooperators select

**Fig 3:**
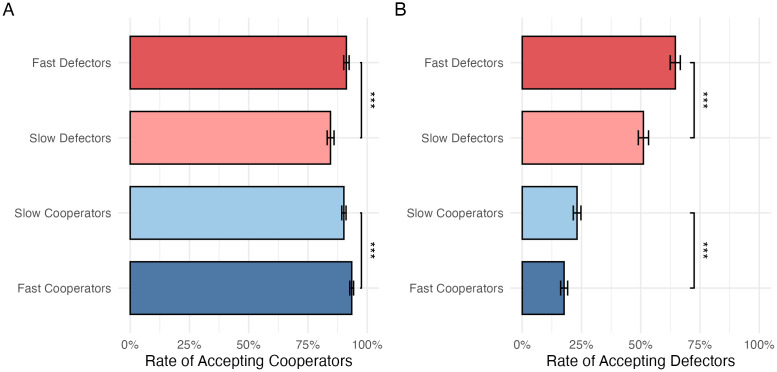
Rates of acceptance among fast and slow participants when offered a chance to connect to (A) a cooperator and (B) a defector. Error bars show 95% CIs of means. ***: *p* < 0.001 in a t-test.

Next, I studied whether decision time predicted acceptance of new neighbors (Fig 3). As discussed above, a rational defector seeking to maximize the benefit should always choose to connect to others when given a chance. However, in the data, when offered a chance to connect to a cooperator (Fig 3A), fast defectors (3,297 out of 3,660, or 90.1%) were also significantly more likely to accept the offer than slow defectors (3,501 out of 4,184, or 83.7%) (*p* < 0.001, two-tailed t-test); when offered a chance to connect to a fellow defector (Fig 3B), fast defectors (1,963 out of 3,143, or 62.5%) were also significantly more likely to accept the offer than slow defectors (1,565 out of 3,115, or 50.2%) (*p* < 0.001, two-tailed t-test).

Fig 3 also shows that, conversely, fast cooperators (702 out of 3,779, or 18.6%) were less likely to accept an offer to connect to a defector than slow cooperators (491 out of 4,065, or 23.1%) (*p* < 0.001, two-tailed t-test), but fast cooperators (4,582 out of 4,956, or 92.5%) were more likely to accept an offer to connect to a fellow cooperator than slow cooperators (5,349 out of 6,042, or 88.5%) (*p* < 0.001, two-tailed t-test).

Likewise, I used binomial GLMMs (Models 2, 3) to control for potential confounders. Here, instead of focusing on the participants who were making the decision, the outcome variables in these two models were the probability to accept an offer to connect to a cooperator and a defector, respectively. Therefore, these models accommodated both defector and cooperator decision makers when predicting the outcomes. The model also controlled for environmental factors, round, and visibility (see S1 Appendix D, E for details).

**Fig 4:**
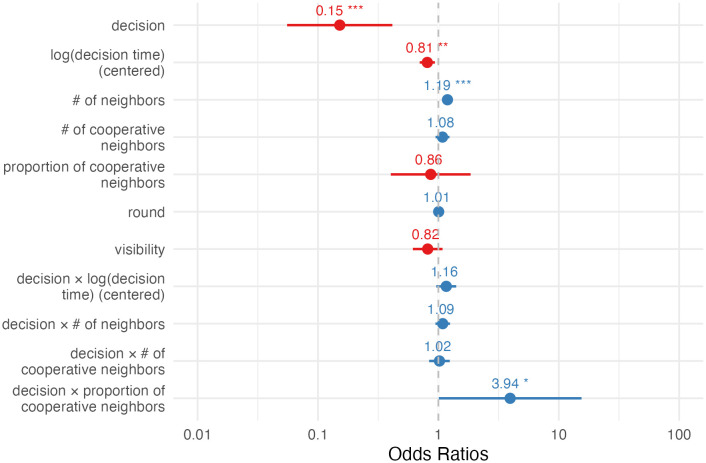
Estimated fixed effects of GLMM predicting offer acceptance (accept = 1) to connect to cooperators (Model 2). Error bars show 95% CI.

Model 2 (Fig 4) reveals the relationship between decision time and the probability to accept a cooperator neighbor. It predicted that a typical defector with one-unit extra log-decision time had a 19% (95% CI = 7%, 30%) decrease in the odds to connect to a cooperator. This means that not all the defectors are the same when it comes to accepting a chance to free ride, and this heterogeneity can be predicted by their feeling of conflict.

However, the model did not support the observation that faster cooperators were more likely to accept fellow cooperators as neighbors than slower ones, after controlling for various potential confounders: For a typical cooperator, an extra unit of log-decision time was associated with a 6% (95% CI=−21%, 27%; var(C)=0.0055, var(D)=0.0095, cov(C,D)=−0.0010, where C and D are log-decision time and the interaction between decision and log-decision time) decrease in the odds to connect to another cooperator.

**Fig 5:**
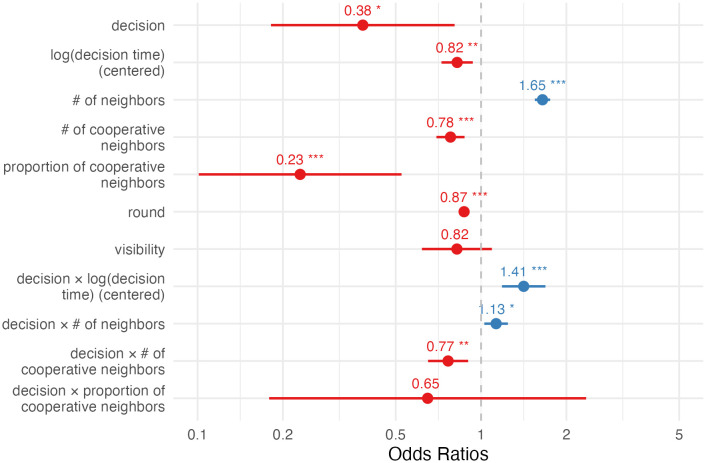
Estimated fixed effects of GLMM predicting offer acceptance (accept = 1) to connect to defectors (Model 3). Error bars show 95% CI.

Model 3 (Fig 5) shows the relationship between decision time and the acceptance of new defector neighbors. For a typical defector, each extra unit of log-decision time was related to an 18% (95% CI = 6%, 28%) decrease in the odds of accepting an offer to connect to another defector.

Model 3 also predicted that, for a typical cooperator, each unit of extra log-decision time was linked with an 16% (95% CI = 1%, 34%; var(C)=0.0042;var(D)=0.0081;cov(C, D)=−0.0037, where D and refer to log-decision time, and the interaction between decision and log-decision time, respectively) increase in the odds of accepting an offer to connect to a defector.

Taken together, the two models show that faster defectors were more eager to connect to others, be them cooperators or defectors, than slower defectors. Meanwhile faster cooperators were less willing to connect to defectors than slower cooperators, but were not significantly different in terms of their willingness to connect to fellow cooperators. Again, models that considered the potential moderation effect of round index supported the above findings, although the positive relationship between decision time and acceptance of new defective neighbors among the cooperators was not significant in the time-varying model (S1 Appendix D, E).

## Discussion

DDM establishes decision time as a measure of mental conflict. It is shown that decision time is indeed useful in the prediction of both participants’ next-round cooperation and social rewiring decisions. In terms of their next-round decisions, the results demonstrate the relationship between the participants’ next-round decisions and their current-round decision time. A longer decision time, which signifies greater mental conflict, predicts a higher likelihood to flip in the next round for both cooperators and defectors. This study also shows the moderating role of decision time in the negative effect on a cooperator’s next-round cooperation of choosing to connect to a defector. In terms of participants’ rewiring choices, the effect of decision time depends on both the ego and the alter’s last decision. Faster defectors are more willing to connect to both defectors and cooperators than slower defectors. Faster cooperators are less willing to connect to a defector than slower cooperators, but there is no significant difference between faster and slower cooperators in terms of their willingness to connect to fellow cooperators. In other words, fast defectors connect, while fast cooperators select.

These results represent an extension to the body of work on the role of decision time in repeated public goods games by extending the relationship between decision time and decision in the same round into different rounds; previously, studies have only linked decision time with the decision in the same round [8–10,26–28]. It is also a further attempt to explicate people’s tie rewiring choices beyond the dichotomy of cooperators and defectors [3] and in different levels of social fluidity [30].

The findings may be useful for devising better intervention strategies that strategically engage defector–cooperator pairs while maintaining transparency, which previous research lacks [5]. This may be useful to remedy the “problem of segregation” induced by the disengagement strategy, which isolates the defecting reciprocators from the cooperative community [4,5,33]. Instead, decision time may be leveraged to identify promising connections to be made between cooperators and defectors, allowing more people to be connected to the “mainstream” community, hereby enhancing collective well-being.

## Supporting information

S1 Appendix (DOCX)

## References

[1] Rand DG, Nowak MA. Human cooperation. Trends Cogn Sci. 2013;17(8):413–25. doi: 10.1016/j.tics.2013.06.003 23856025

[2] Axelrod RM. The evolution of cooperation. New York: Basic Books. 1984.

[3] Rand DG, Arbesman S, Christakis NA. Dynamic social networks promote cooperation in experiments with humans. Proc Natl Acad Sci U S A. 2011;108(48):19193–8. doi: 10.1073/pnas.1108243108 22084103 PMC3228461

[4] Shirado H, Christakis NA. Network Engineering Using Autonomous Agents Increases Cooperation in Human Groups. iScience. 2020;23(9):101438. doi: 10.1016/j.isci.2020.101438 32823053 PMC7452167

[5] McKee KR, Tacchetti A, Bakker MA, Balaguer J, Campbell-Gillingham L, Everett R, et al. Scaffolding cooperation in human groups with deep reinforcement learning. Nat Hum Behav. 2023;7(10):1787–96. doi: 10.1038/s41562-023-01686-7 37679439 PMC10593606

[6] Krajbich I, Armel C, Rangel A. Visual fixations and the computation and comparison of value in simple choice. Nat Neurosci. 2010;13(10):1292–8. doi: 10.1038/nn.2635 20835253

[7] Fudenberg D, Newey W, Strack P, Strzalecki T. Testing the drift-diffusion model. Proceedings of the National Academy of Sciences. 2020;117:33141–8. doi: 10.1073/pnas.2011446117 PMC7776861 33310903

[8] Evans AM, Dillon KD, Rand DG. Fast but not intuitive, slow but not reflective: Decision conflict drives reaction times in social dilemmas. J Exp Psychol Gen. 2015;144(5):951–66. doi: 10.1037/xge0000107 26413891

[9] Evans AM, Rand DG. Cooperation and decision time. Curr Opin Psychol. 2019;26:67–71. doi: 10.1016/j.copsyc.2018.05.007 29879640

[10] Nishi A, Christakis NA, Evans AM, O’Malley AJ, Rand DG. Social Environment Shapes the Speed of Cooperation. Scientific Reports. 2016;6:29622. doi: 10.1038/srep29622 27435940 PMC4951649

[11] Camerer C. Behavioral game theory: experiments in strategic interaction. New York: Princeton, N.J.: Russell Sage Foundation; Princeton University Press; 2003.

[12] Marwell G, Ames RE. Experiments on the provision of public goods. I. Resources, interest, group size, and the free-rider problem. Am J Sociol. 1979;84:1335–60.

[13] Suri S, Watts DJ. Cooperation and contagion in web-based, networked public goods experiments. PLoS One. 2011;6(3):e16836. doi: 10.1371/journal.pone.0016836 21412431 PMC3055889

[14] Shirado H. Autonomous-agent interventions in networked human cooperation and coordination. Yale University. 2019. https://www.proquest.com/dissertations-theses/autonomous-agent-interventions-networked-human/docview/2304965018/se-2?accountid=15172

[15] Olson M. The logic of collective action: public goods and the theory of groups. Cambridge, Mass: Harvard University Press. 1971.

[16] Fowler JH, Christakis NA. Cooperative behavior cascades in human social networks. Proc Natl Acad Sci U S A. 2010;107(12):5334–8. doi: 10.1073/pnas.0913149107 20212120 PMC2851803

[17] Centola D, Macy M. Complex Contagions and the Weakness of Long Ties. American Journal of Sociology. 2007;113(3):702–34. doi: 10.1086/521848

[18] Bavard S, Stuchlý E, Konovalov A, Gluth S. Humans can infer social preferences from decision speed alone. PLoS Biol. 2024;22(6):e3002686. doi: 10.1371/journal.pbio.3002686 38900903 PMC11189591

[19] Konovalov A, Krajbich I. Decision times reveal private information in strategic settings: evidence from bargaining experiments. Rochester, NY: Social Science Research Network. 2020. doi: 10.2139/ssrn.3023640

[20] Konovalov A, Krajbich I. Revealed strength of preference: Inference from response times. Judgm decis mak. 2019;14(4):381–94. doi: 10.1017/s1930297500006082

[21] Jordan JJ, Hoffman M, Nowak MA, Rand DG. Uncalculating cooperation is used to signal trustworthiness. Proc Natl Acad Sci U S A. 2016;113(31):8658–63. doi: 10.1073/pnas.1601280113 27439873 PMC4978259

[22] Konovalov A, Krajbich I. On the strategic use of response times. SSRN Journal. 2017. doi: 10.2139/ssrn.3023640

[23] Milosavljevic M, Malmaud J, Huth A, Koch C, Rangel A. The Drift Diffusion Model can account for the accuracy and reaction time of value-based choices under high and low time pressure. Judgm decis mak. 2010;5(6):437–49. doi: 10.1017/s1930297500001285

[24] Ratcliff R, McKoon G. The diffusion decision model: theory and data for two-choice decision tasks. Neural Comput. 2008;20(4):873–922. doi: 10.1162/neco.2008.12-06-420 18085991 PMC2474742

[25] Krajbich I, Rangel A. Multialternative drift-diffusion model predicts the relationship between visual fixations and choice in value-based decisions. Proc Natl Acad Sci U S A. 2011;108(33):13852–7. doi: 10.1073/pnas.1101328108 21808009 PMC3158210

[26] Krajbich I, Bartling B, Hare T, Fehr E. Rethinking fast and slow based on a critique of reaction-time reverse inference. Nat Commun. 2015;6:7455. doi: 10.1038/ncomms8455 26135809 PMC4500827

[27] Lotito G, Migheli M, Ortona G. Is cooperation instinctive? Evidence from the response times in a public goods game. J Bioecon. 2013;15:123–33. doi: 10.1007/s10818-012-9141-5

[28] Nishi A, Christakis NA, Rand DG. Cooperation, decision time, and culture: Online experiments with American and Indian participants. PLoS One. 2017;12(2):e0171252. doi: 10.1371/journal.pone.0171252 28231296 PMC5322955

[29] Nishi A, Shirado H, Rand DG, Christakis NA. Inequality and visibility of wealth in experimental social networks. Nature. 2015;526(7573):426–9. doi: 10.1038/nature15392 26352469

[30] Shirado H, Fu F, Fowler JH, Christakis NA. Quality versus quantity of social ties in experimental cooperative networks. Nat Commun. 2013;4:2814. doi: 10.1038/ncomms3814 24226079 PMC3868237

[31] McKnight ME, Christakis NA. Breadboard: Software for Online Social Experiments. Yale University. 2016. https://breadboard.yale.edu/

[32] Rand DG, Greene JD, Nowak MA. Spontaneous giving and calculated greed. Nature. 2012;489(7416):427–30. doi: 10.1038/nature11467 22996558

[33] Kurzban R, Houser D. Experiments investigating cooperative types in humans: a complement to evolutionary theory and simulations. Proc Natl Acad Sci U S A. 2005;102(5):1803–7. doi: 10.1073/pnas.0408759102 15665099 PMC547861

