# Supplementary material for: Decision time is associated with future cooperation and social rewiring decisions in network public goods games
Source: PLoS One. 2026 Apr 28;21(4):e0347919. doi: 10.1371/journal.pone.0347919 (PMC13123959; doi:10.1371/journal.pone.0347919)
Supplement: S1 Appendix — (DOCX) [file pone.0347919.s001.docx]

**S1 Appendix**

Appendix A: overall distribution of log-decision time and distributions of log-decision time over time

Fig A shows the overall log-transformed decision time distribution of each decision made by every participant in every round (left) and the log-decision time distributions of every participant’s decision over the rounds (right). These distributions are pooled over the sessions. It can be seen that the raw decision time distribution was highly skewed and the participants became faster making the cooperation decisions over time.

| 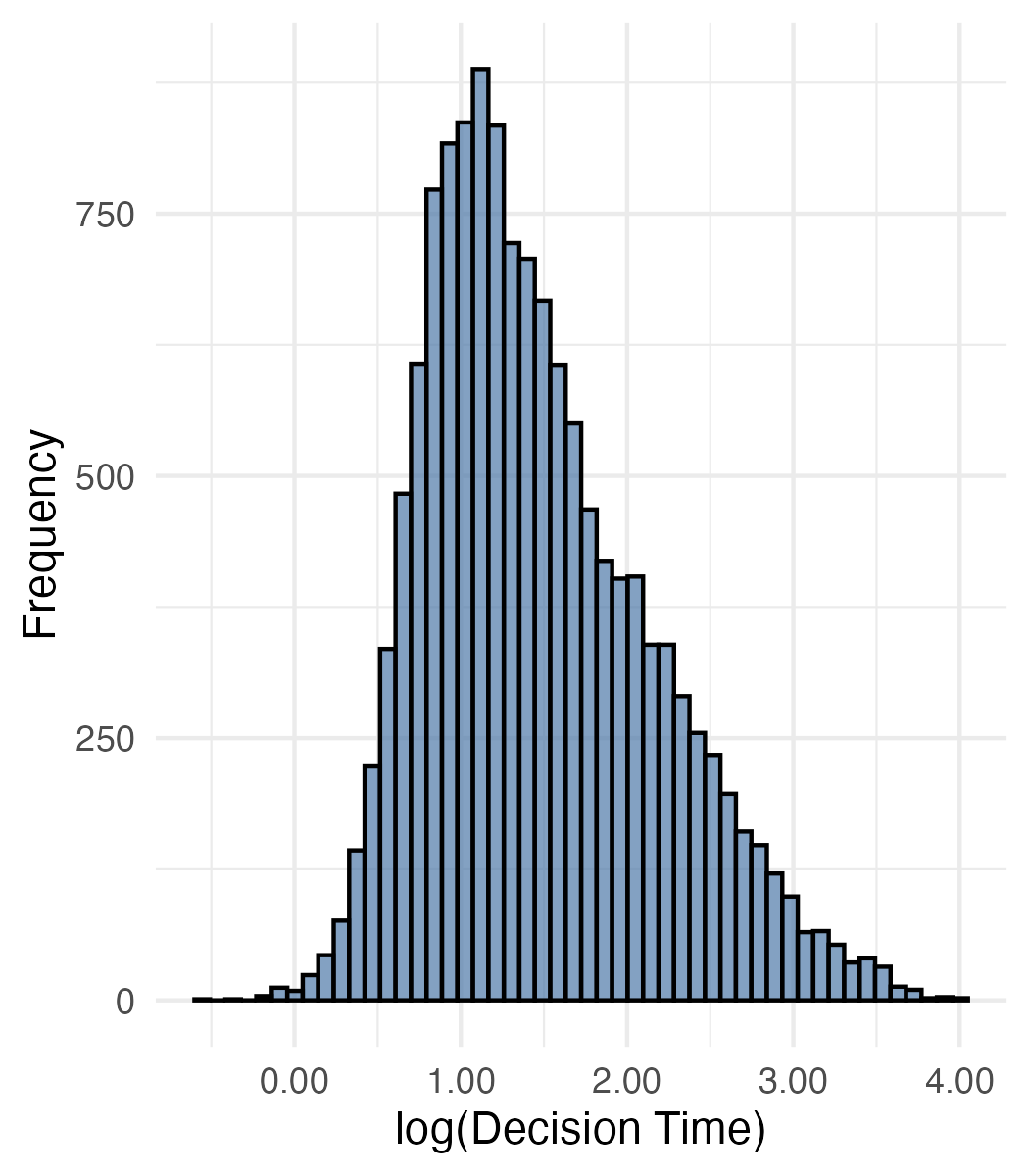 | 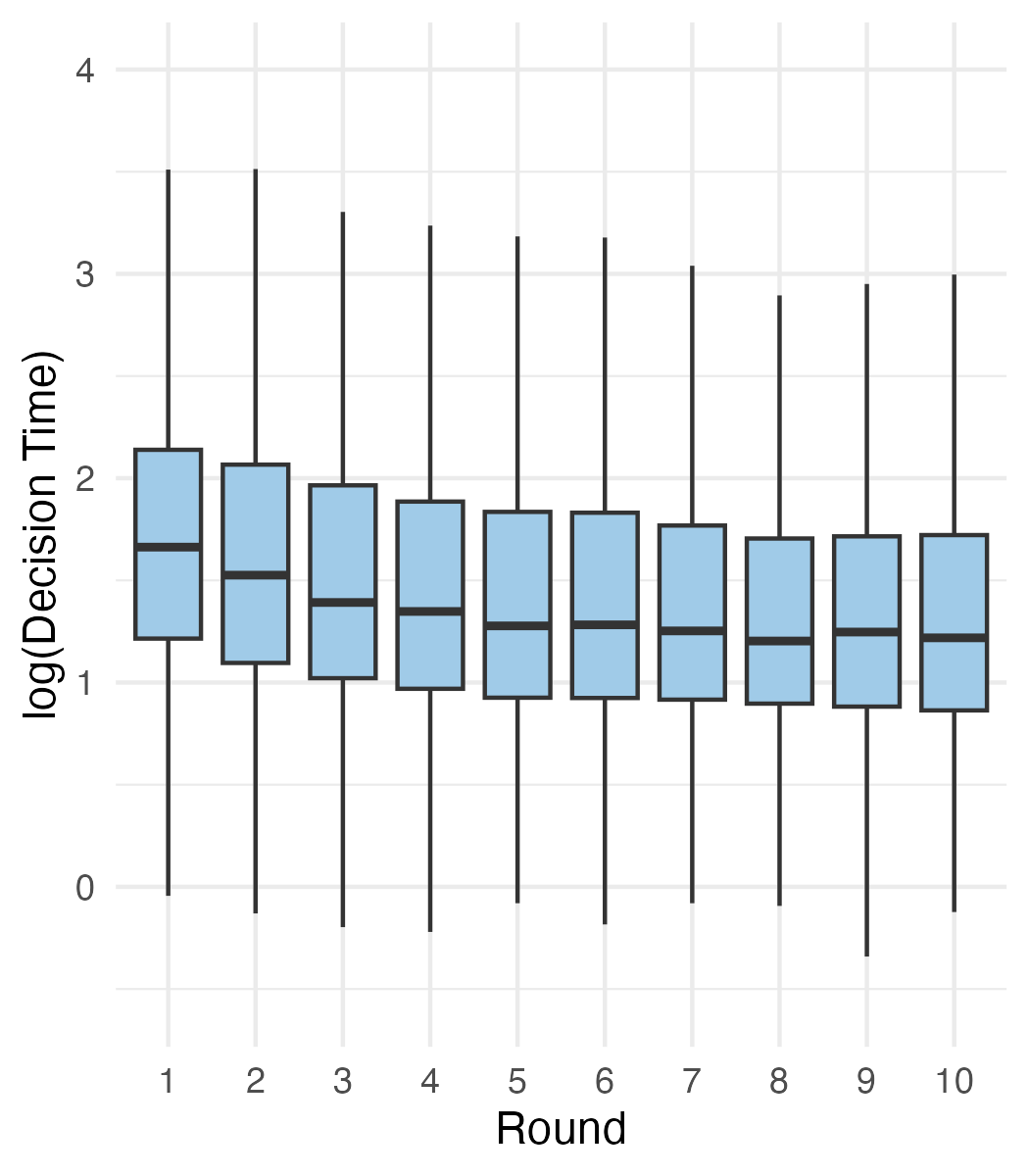 |
| --- | --- |
| 1. Distribution of log-transformed decision time of every participant–round | 1. Distributions of log-transformed decision time in each round |

**Fig A. Distribution of log-decision time pooled over the rounds and distributions of log-decision time in each round**.

Appendix B: Next-round cooperation when participants are offered, accepted new connections and when the connections are established

Fig B shows the next-round cooperation rates (a) when a participant (stratified by decision) was offered a chance to connect to at least one other participant (also stratified by decision), (b) when they accepted those offers, and (c) when the connection was successfully established (as it was also contingent upon the other participant’s acceptance of the offer). Again, the “fast” refer to those whose decision times were in the bottom 50% and the “slow” refer to those whose decision times were in the top 50% (pooled).

| 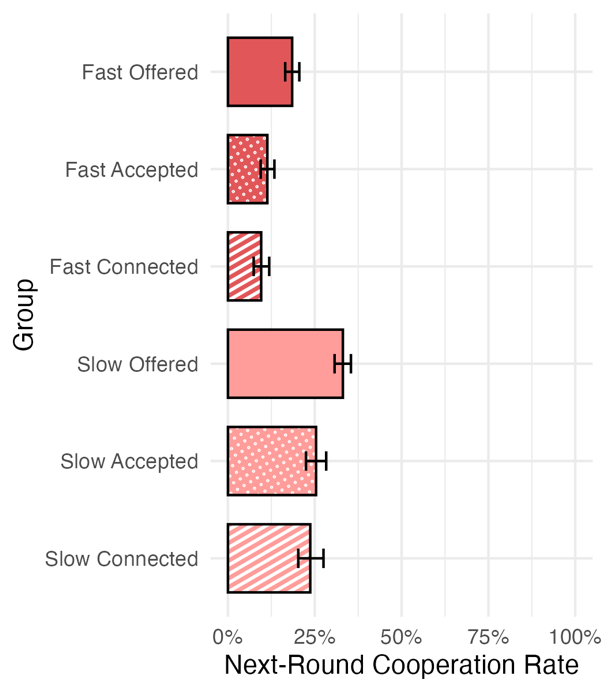 | 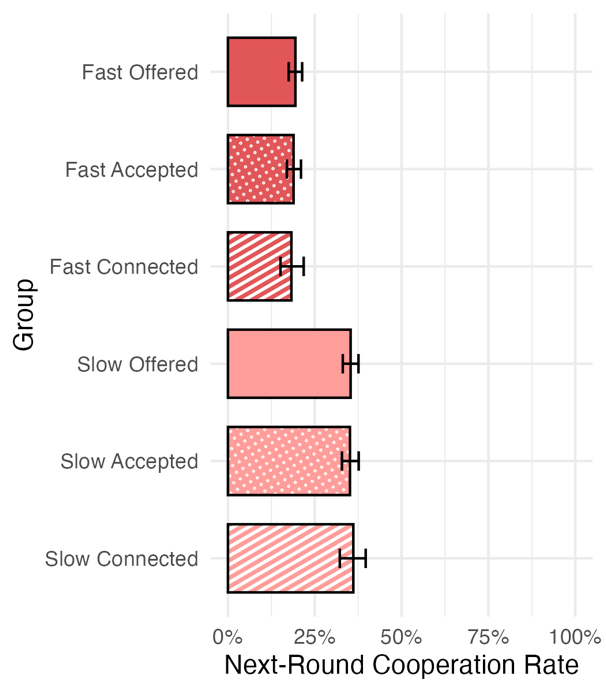 |
| --- | --- |
| 1. Defectors’ next-round decisions when offered to connect to defectors | 1. Defectors’ next-round decisions when offered to connect to cooperators |
| 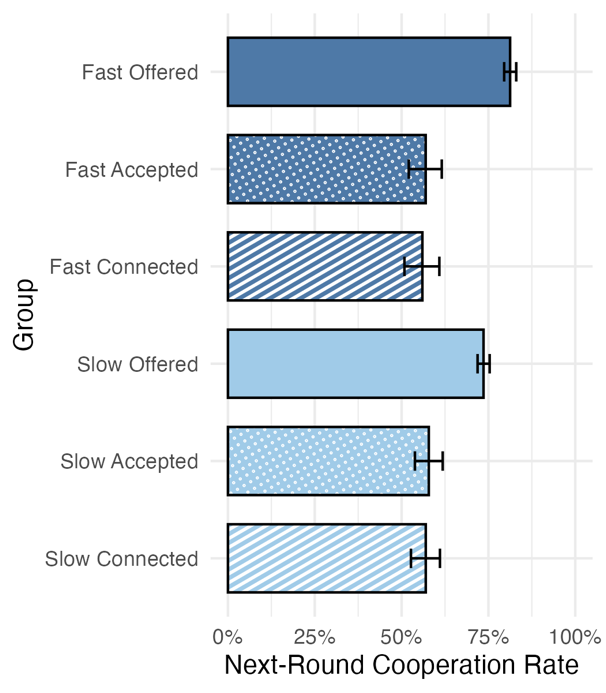 | 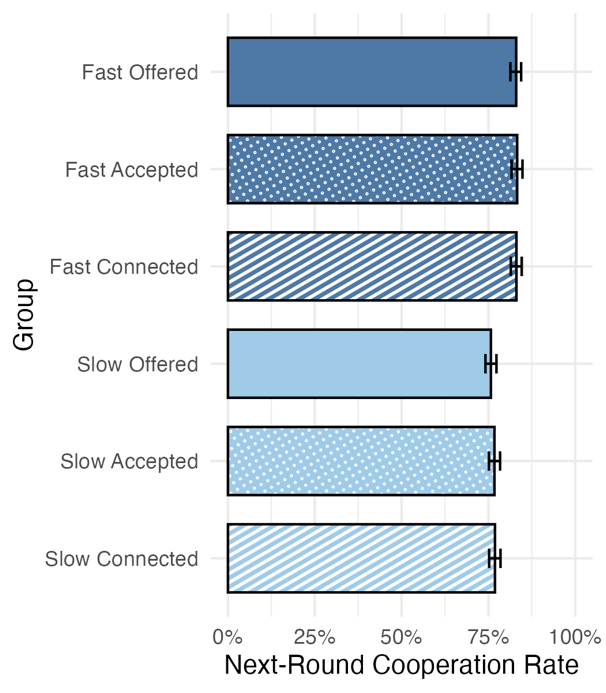 |
| 1. Cooperators’ next-round decisions when offered to connect to defectors | 1. Cooperators’ next-round decisions when offered to connect to cooperators |

**Fig B. Next-round cooperation rates of participants who were offered a chance to connect to another participant, who accepted the offer, and who successfully made the connection(s).** Error bars show 95% CI.

For defectors getting connected to defectors, fast defectors who were offered such a chance were significantly more likely to cooperate in the next round than those who accepted this offer (*p*<0.001, two-tailed t-test). There was no significant difference between those who accepted such an offer and those who ended up had these connections established (*p*=0.247, two-tailed t-test). Likewise, slow defectors who were offered such a chance were significantly more likely to cooperate in the next round than those who accepted this offer (*p*<0.001, two-tailed t-test). There was no significant difference between those who accepted such an offer and those who ended up had these connections established (*p*=0.469, two-tailed t-test).

For defectors getting connected to cooperators, there were no significant differences among the three conditions within the fast and slow ones, respectively (*p*=0.708, 0.754, 0.560, two-tailed t-tests for FO vs. FA, FA vs. FC, and FO vs. FC; *p*=0.911, 0.677, 0.733, two-tailed t-tests for SO vs. SA, SA vs. SC, and SO vs. SC; F=Fast, S=Slow, O=Offered, A=Accepted, C=Connected). In other words, when a defector was offered a chance to connect to a cooperator, whether the defector accepted the offer and if they did, whether the tie was eventually made (which also depended on the cooperator’s acceptance), did not predict the defector’s next-round decision. Therefore, in a model that predicts defectors’ next-round decision with information about rewiring (Model 1), it should not make a difference whether one chooses to use the number of offers, the number of accepted offers, or the number of established connections as the predictor.

For cooperators getting connected to defectors, there was a difference between the fast ones and the slow ones when offered a chance to connect to a defector. As expected, the fast ones were more likely to cooperate in the next round than the slow ones when offered to connect to a defector (*p*<0.001, two-tailed t-test). However, when a fast cooperator accepted such an offer, they would experience a greater decrease in their probability to cooperate next round than a slow cooperator, resulting in a statistically insignificant difference between their next-round cooperation rate (*p*=0.777, two-tailed t-test). Again, there were no significant differences between an accepted offer and an established connection for fast (*p*=0.789, two-tailed t-test) and slow cooperators (*p*=0.767, two-tailed t-test). For cooperators, this might be because once they agreed to connecting to a defector, this connection would be highly likely to be made, since the defector on the other end of this tie would usually agree to the connection.

For cooperators getting connected to cooperators, there were no significant differences among the three conditions within the fast and slow ones, respectively (*p*=0.794, 0.848, 0.949, two-tailed t-tests for FO vs. FA, FA vs. FC, and FO vs. FC; *p*=0.378, 0.900, 0.319, two-tailed t-tests for SO vs. SA, SA vs. SC, and SO vs. SC).

In sum, for both defectors and cooperators, fast and slow, there were no significant differences between accepting an offer and having the connection realized in terms of their next-round cooperation rates. Meanwhile, accepting offers to connect to defectors were associated with lowered next-round cooperation rate compared with merely being offered such a chance. Therefore, it was necessary to use the acceptance of offers to predict next-round cooperation decision, not the offers themselves, which directly informed the specification of Model 1.

Appendix C: glmm predicting next-round cooperation (model 1)

The estimated fixed effects (and their 95% CIs) of Model 1 are summarized in Fig 2. The model incorporated a random intercept for each participant, resulting in a 1.43 standard deviation of this random effect.

Apart from the effects of decision and decision time, this model also showed that for a typical participant, the next-round decision is negatively related to the number of neighbors and positively related to the number of cooperative neighbors and the proportion of cooperative neighbors. This is consistent with previous theoretical discussions [1] as well as empirical observations [2]. In addition, the negative effects of round and visibility are also consistent with existing literature [2,3].

The model also revealed how the decisions to accept an offer to connect to defectors and cooperators predicted one’s next-round decision, as alluded to in Appendix B. For a typical defector, connecting to an extra defector predicted a 27% (95% CI=18%, 34%) decrease in the odds of cooperating next round, and connecting to an extra cooperator predicted a 11% (95% CI=5%, 17%) decrease in the odds of cooperating next round, after controlling for various factors in the model. For a typical cooperator, connecting to an extra defector predicted a 31% (95% CI=22%, 38%) decrease, and connecting to an extra cooperator predicted an 14% (95% CI=9%, 19%) decrease ($\mathrm{var}\left( E \right)=0.0031;var\left( F \right)=0.006;cov\left( E, F \right)=-0.0028;var\left( G \right)=0.0012;var\left( H \right)=0.0018;cov\left( G, H \right)=-0.0010$, where $E, F, G, H$ means choosing to connect to an extra defector, the interaction between decision and choosing to connect to an extra defector, choosing to connect to an extra cooperator, the interaction between decision and choosing to connect to an extra cooperator, respectively).

Meanwhile, when predicting the outcome, one should also note that connecting to a new neighbor would change the environmental factors, which, in turn, would change the participant’s probability of cooperation in the next round. Of course, this would be dependent on the alter’s acceptance. For instance, choosing to connect to a cooperator would result in an increase in the number of neighbors, which would have a negative effect, and the number of cooperative neighbors, which would have a positive effect, and the proportion of cooperative neighbors, which would also have a positive effect. The total effect would typically be positive because the positive effect of getting an extra cooperative neighbor outweighs the negative effect of getting an extra neighbor and getting an extra cooperative neighbor would never decrease the proportion of cooperative neighbors among all one’s neighbors, although the exact effect size would depend on this proportion in the given round.

Here, I offer a more sophisticated model that incorporated the time-varying effect of decision time.^[[1]](#footnote-1)^ To be specific, I used centered round index instead of raw round index in the new model. I included three extra terms in this model: decision × round, log-decision time × round, and decision × log-decision time × round. This allows one to compute the conditional effect of decision time for each decision category (cooperator and defector) in every round.

The fitted fixed effects are summarized in the figure below. While there was a significant interaction between decision and round, meaning that the carry-over effect was weakening for cooperators, no time-varying effects were observed of decision time on next-round cooperation, evidenced by the insignificant coefficients of the two-way interaction between decision time and round as well as and the three-way interaction among decision, decision time, and round.


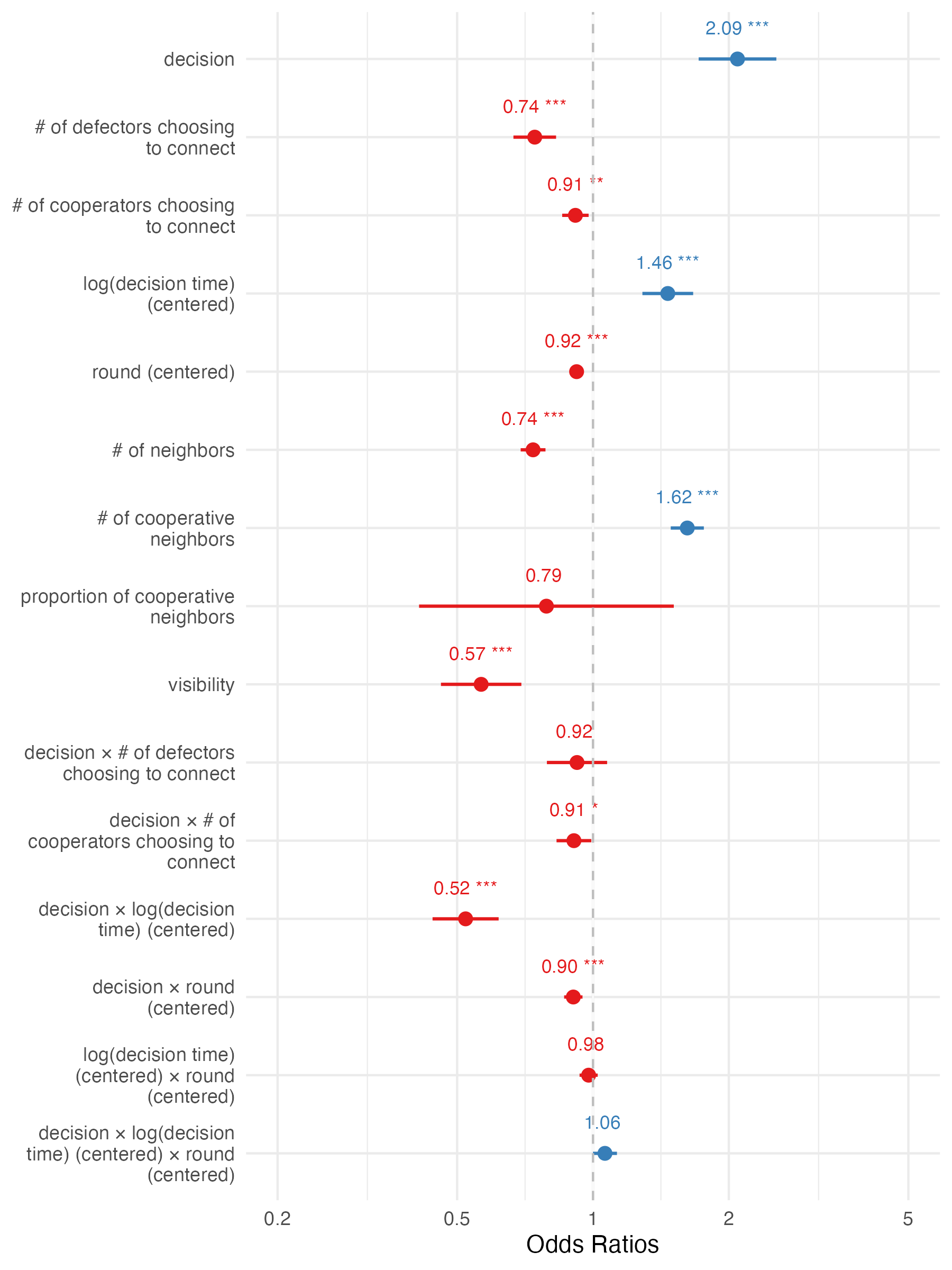


**Fig C. Estimated fixed effects of the updated Model 1, which considers time-varying effects of decision time, predicting next-round cooperation (cooperate=1).** Error bars show 95% CI.

To compute the conditional effects of cooperators and defectors, I used the appropriate linear combinations of the coefficients above and hypothetical round indices. The effects are summarized in the figure below, with shaded areas denoting 95% confidence intervals. Visually, the effect of decision time on next-round cooperation was declining for both cooperators and defectors. However, as discussed above, these visual trends were not statistically significant. To conclude, this more sophisticated model supported the findings from the original Model 1.


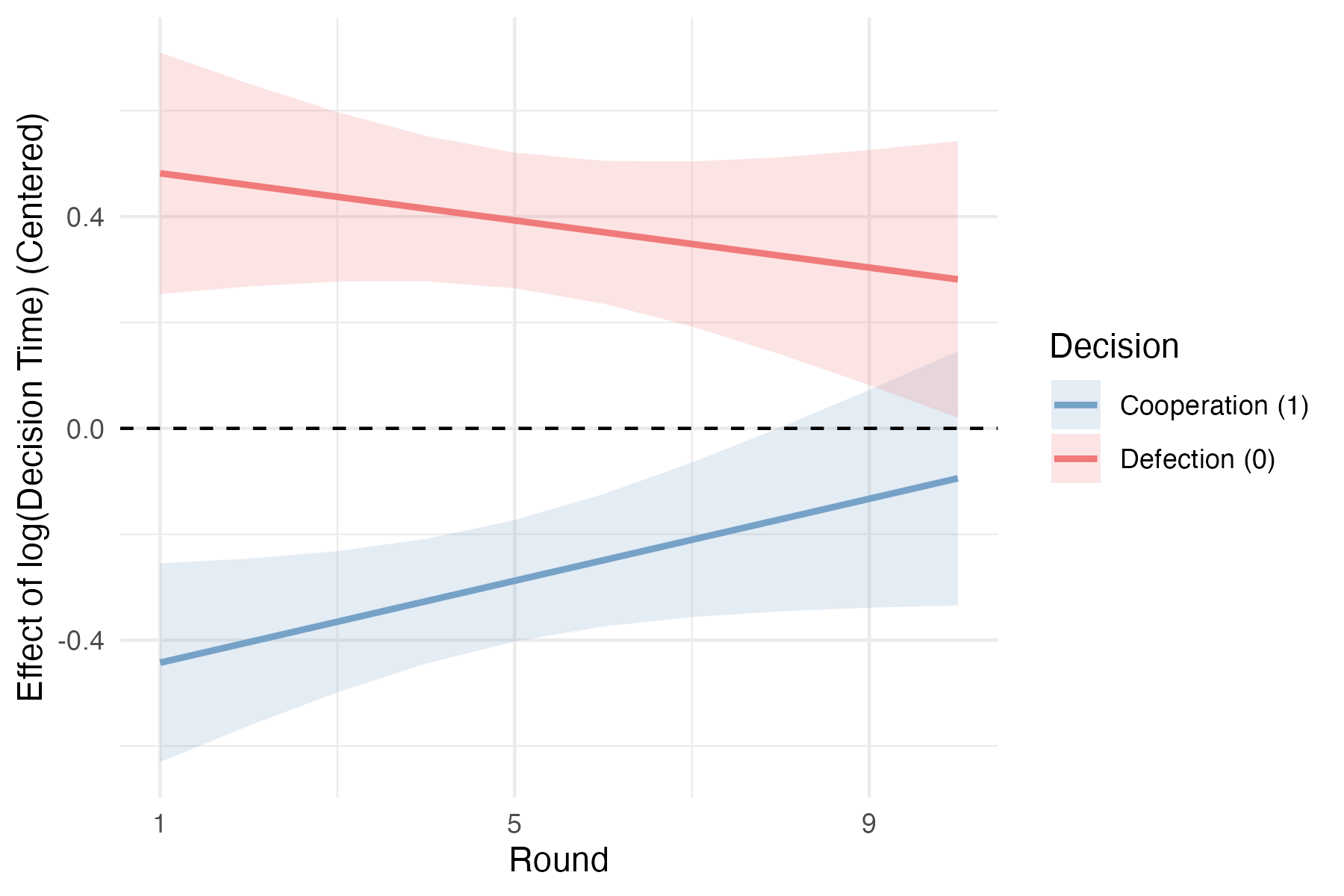


**Fig D. Estimated conditional effects of log-decision time on next-round cooperation (cooperate=1) for cooperators and defectors in each round.** Error bars show 95% CI.

Appendix D: binomial glmm predicting probability of accepting offers to connect to cooperators (model 2)

The estimated fixed effects (and their 95% CIs) of Model 2 are summarized in Figure 4. The model incorporated a random intercept for each participant, resulting in a 1.96 standard deviation of this random effect.

This model showed that for a typical participant, their willingness to connect to a cooperator is positively predicted by their current number of neighbors. One should be cautious when interpreting this result, as a high current number of neighbors can be a result of high willing to connect (with cooperators or defectors).

Again, a robustness check was conducted by including the same three interaction terms as for Model 1 to allow for time-varying effects. The fixed effects are shown below. For defectors, the negative effect of decision time on accepting cooperators grew significantly stronger over time.


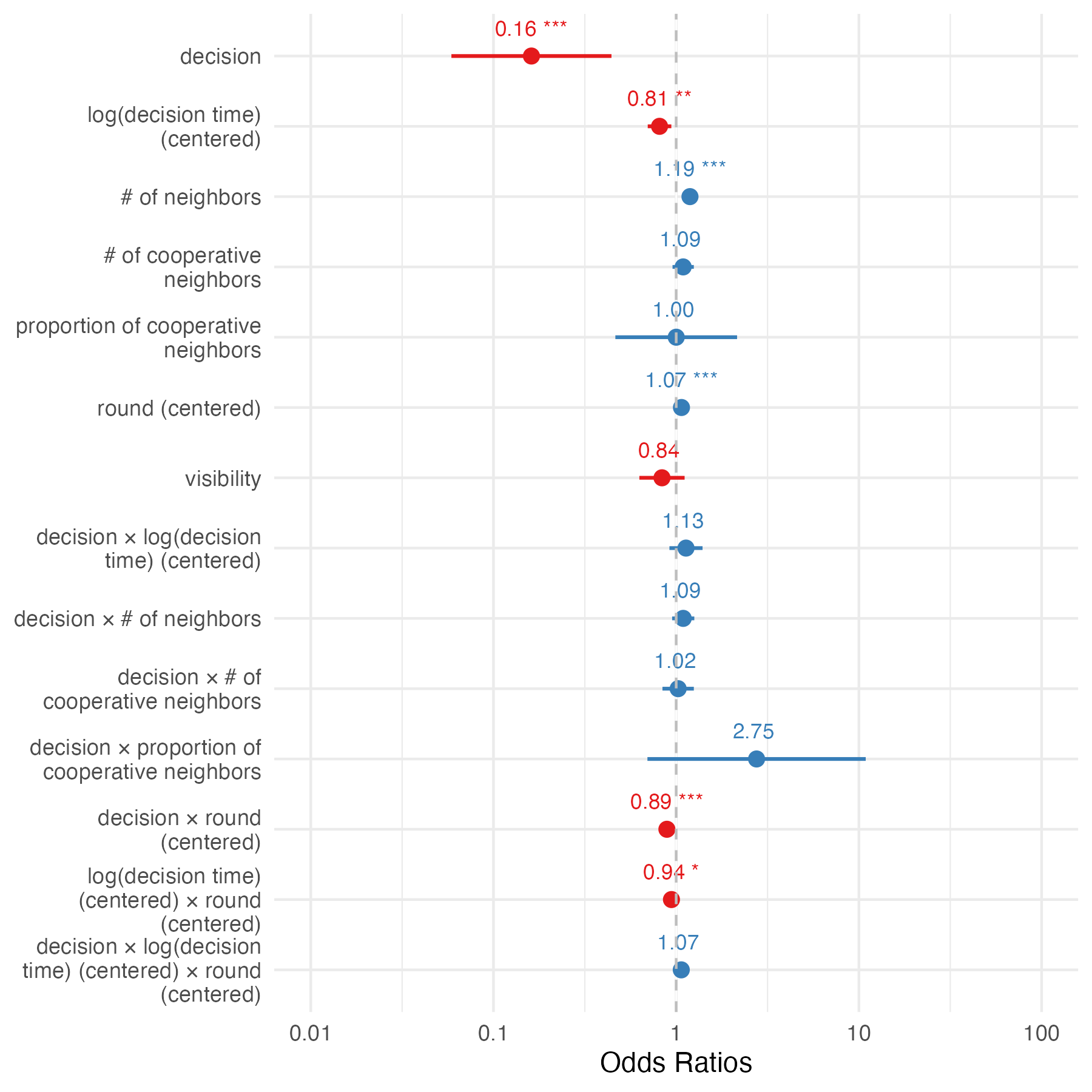


**Fig E. Estimated fixed effects of GLMM predicting offer acceptance (accept=1) to connect to cooperators while considering time-varying effects of decision time.** Error bars show 95% CI.

Again, I visualized the conditional effects of decision time on accepting offers to connect to a cooperator. As is easier to see in this figure, the negative relationship between decision time and accepting cooperators for defectors was less pronounced in earlier rounds and more pronounced in later rounds. For cooperators, the effect was not statistically significant. Overall, this model still supported the findings from the original model.


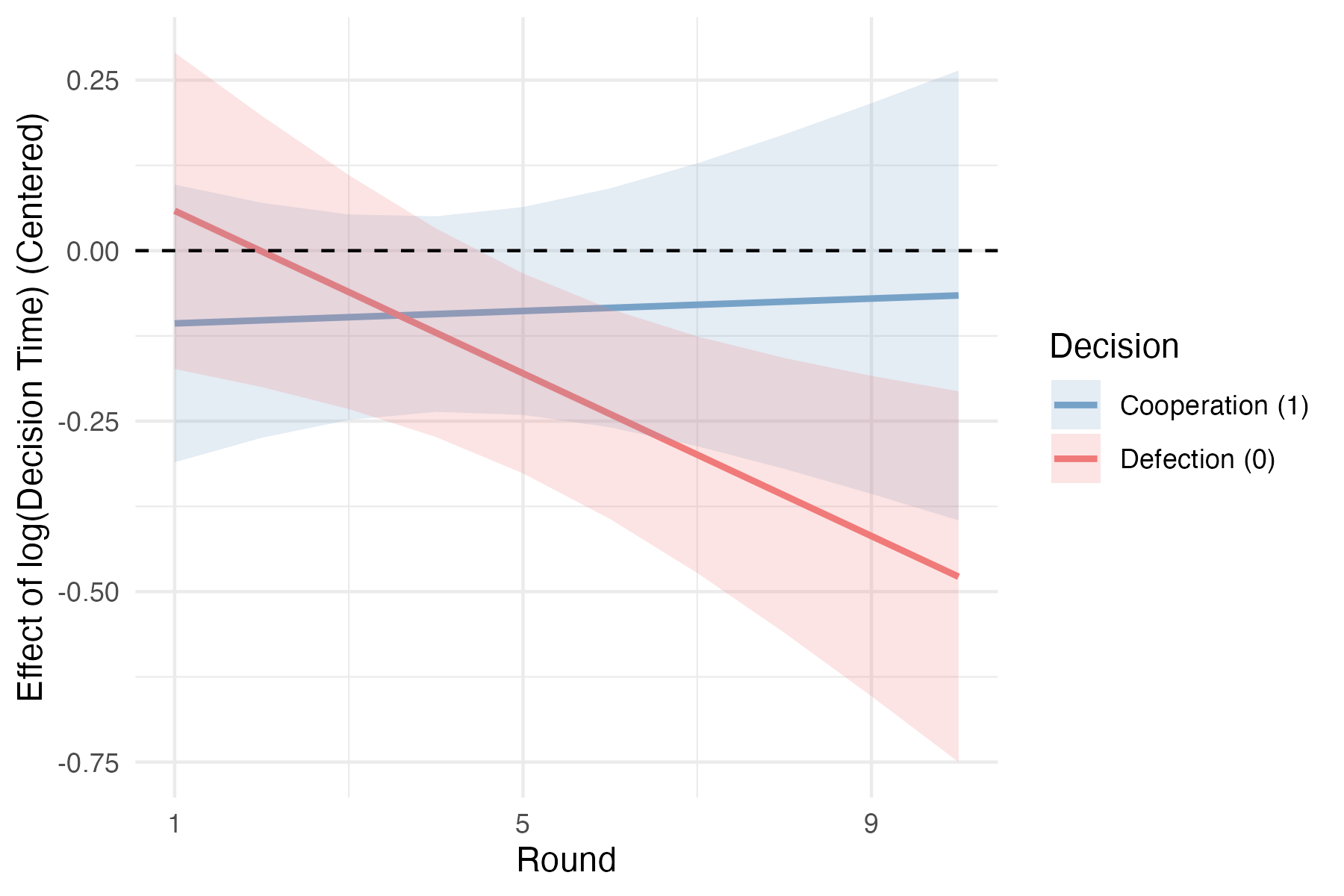


**Fig F. Estimated conditional effects of log-decision time on offer acceptance (accept=1) to connect to cooperators for cooperators and defectors in each round.** Error bars show 95% CI.

Appendix E: binomial glmm predicting probability of accepting offers to connect to DEFECTORS (model 3)

The estimated fixed effects (and their 95% CIs) of Model 3 are summarized in Figure 5. The model incorporated a random intercept for each participant, resulting in a 2.14 standard deviation of this random effect.

This model showed an interesting picture—no matter a defector or a cooperator, a typical participant was more likely to accept an offer to connect to a defector when they had more neighbors, fewer cooperative neighbors, and a smaller proportion of cooperative neighbors in the neighborhood. For defectors, this could be explained by a free-riding mindset. A “die-hard” free rider would try to connect to whoever, since they had nothing to lose but their neighbors may turn to cooperation, no matter how unlikely. These people usually had more neighbors, fewer cooperative neighbors, and a lower fraction of cooperative neighbors.

These effects were in the same directions but even more pronounced for cooperators than for defectors. This showed that those who had a highly cooperative social environment might be less willing to change their environment.

Again, a time-varying model was built upon this model, the fixed effects of which are summarized below. One can observe the same strengthening negative effect of decision time on accepting fellow defectors for defectors, evidenced by the negative, significant coefficient of the interaction between decision time and round index.


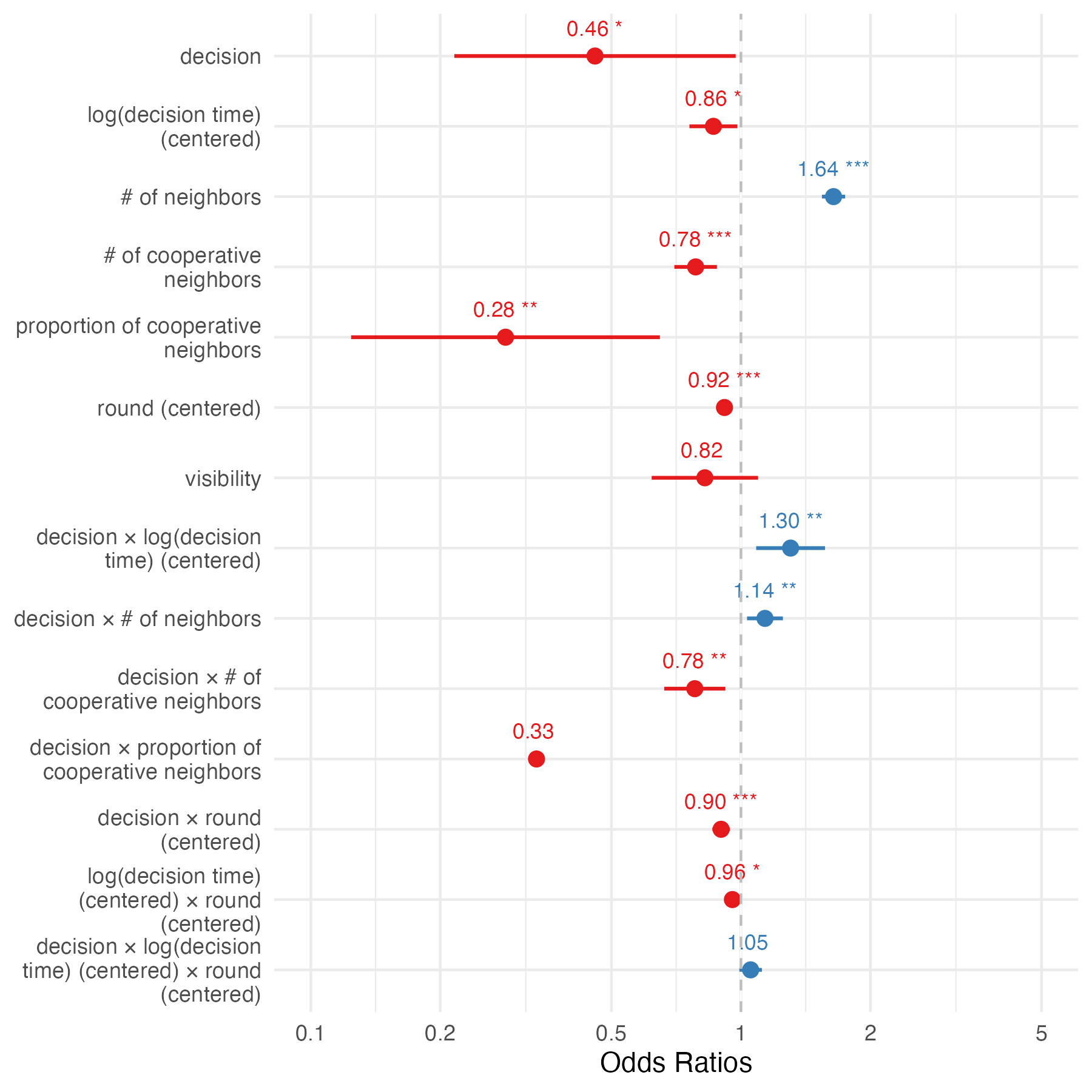


**Fig G. Estimated fixed effects of GLMM predicting offer acceptance (accept=1) to connect to defectors while considering time-varying effects of decision time.** Error bars show 95% CI.

The conditional effects here confirmed the original picture for defectors—in most rounds, decision time had a negative effect on accepting defector neighbors. For cooperators, although the relationship was still positive, as predicted by the original model, it was not significant. This does not contradict the original findings, though, because for cooperators, there was no evidence for time-varying effects, and the additional complexity introduced by the new model was unnecessary, harming the power of the model. Therefore, I conclude that the original findings from Model 3 were supported.^[[2]](#footnote-2)^


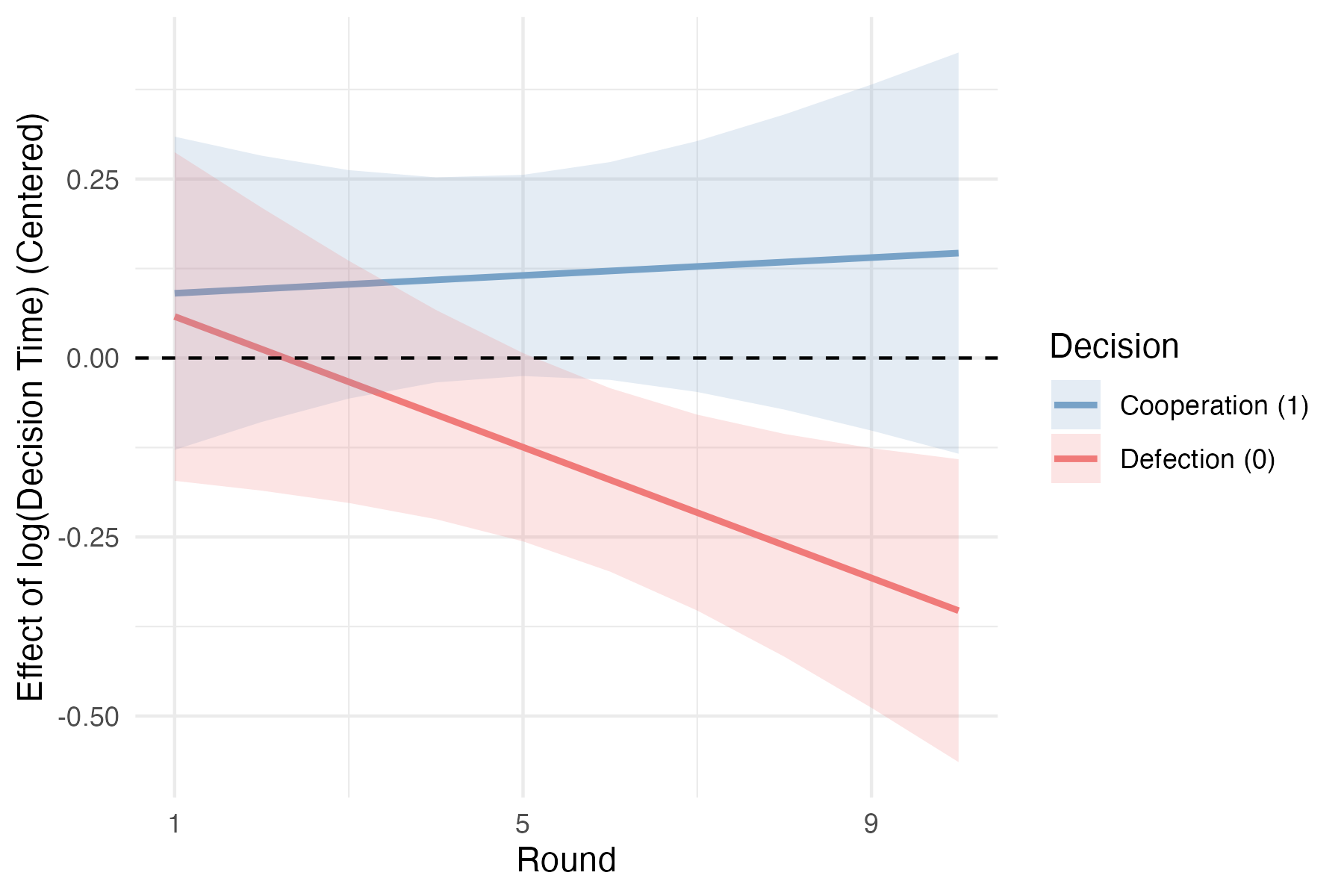


**Fig H. Estimated conditional effects of log-decision time on offer acceptance (accept=1) to connect to defectors for cooperators and defectors in each round.** Error bars show 95% CI.

**References**

1. Shirado H. Autonomous-Agent Interventions in Networked Human Cooperation and Coordination. ProQuest Dissertations and Theses. Ph.D., Yale University. 2019. Available: https://www.proquest.com/dissertations-theses/autonomous-agent-interventions-networked-human/docview/2304965018/se-2?accountid=15172

2. Shirado H, Christakis NA. Network Engineering Using Autonomous Agents Increases Cooperation in Human Groups. iScience. 2020;23: 101438. doi:10.1016/j.isci.2020.101438

3. Nishi A, Shirado H, Rand DG, Christakis NA. Inequality and visibility of wealth in experimental social networks. Nature. 2015;526: 426–429. doi:10.1038/nature15392

1. I am indebted to a reviewer for suggesting this and the following time-varying models in Appendices D and E. [↑](#footnote-ref-1)
2. Note that according to the original model, the 95% CI of this effect for cooperators was 1%, 34%. [↑](#footnote-ref-2)
